# Computing Entropy for Long-Chain Alkanes Using Linear Regression: Application to Hydroisomerization

**DOI:** 10.3390/e26121120

**Published:** 2024-12-21

**Authors:** Shrinjay Sharma, Richard Baur, Marcello Rigutto, Erik Zuidema, Umang Agarwal, Sofia Calero, David Dubbeldam, Thijs J. H. Vlugt

**Affiliations:** 1 Engineering Thermodynamics, Process & Energy Department, Faculty of Mechanical Engineering, Delft University of Technology, Leeghwaterstraat 39, 2628 CB Delft, The Netherlands; s.sharma-6@tudelft.nl; 2 Shell Global Solutions International B.V., Grasweg 39, 1031 HW Amsterdam, The Netherlands; richard.baur@shell.com (R.B.); marcello.rigutto@shell.com (M.R.); erik.zuidema@shell.com (E.Z.); 3 Shell Chemical LP, Monaca, PA 15061, USA; u.agarwal@shell.com; 4 Department of Applied Physics, Eindhoven University of Technology, 5600 MB Eindhoven, The Netherlands; s.calero@tue.nl; 5 Van ’t Hoff Institute of Molecular Sciences, University of Amsterdam, Science Park 904, 1098 XH Amsterdam, The Netherlands; d.dubbeldam@uva.nl

**Keywords:** entropy, linear regression, alkanes, hydroisomerization

## Abstract

Entropies for alkane isomers longer than C_10_ are computed using our recently developed linear regression model for thermochemical properties which is based on second-order group contributions. The computed entropies show excellent agreement with experimental data and data from Scott’s tables which are obtained from a statistical mechanics-based correlation. Entropy production and heat input are calculated for the hydroisomerization of C_7_ isomers in various zeolites (FAU-, ITQ-29-, BEA-, MEL-, MFI-, MTW-, and MRE-types) at 500 K at chemical equilibrium. Small variations in these properties are observed because of the differences in reaction equilibrium distributions for these zeolites. The effect of chain length on heat input and entropy production is also studied for the hydroisomerization of C_7_, C_8_, C_10_, and C_14_ isomers in MTW-type zeolite at 500 K. For longer chains, both heat input and entropy production increase. Enthalpies and absolute entropies of C_7_ hydroisomerization reaction products in MTW-type zeolite increase with higher temperatures. These findings highlight the accuracy of our linear regression model in computing entropies for alkanes and provide insight for designing and optimizing zeolite-catalyzed hydroisomerization processes.

## 1. Introduction

Entropy data are important for a wide range of hydrocarbon applications, from industrial processes [1] to environmental assessments [2]. Entropy changes during chemical reactions like hydroisomerization, combustion, and cracking have a large influence on the feasibility of such reactions [3]. Iso-alkanes with high branching are preferred over linear ones in sustainable aviation fuel and lubricants [4], making shape-selective zeolite-catalyzed hydroisomerization [5,6], also known as catalytic dewaxing, important for the production of these alkanes [7,8]. Understanding thermochemical properties, such as entropies and enthalpies, is essential for designing efficient processes and equipment for (iso)alkane handling and separation [9]. Process energy efficiency is best evaluated through the second law of thermodynamics [3,10]. The second law efficiency requires knowledge on exergy destruction which is the product of entropy production and the temperature of the environment [3,10]. Exergy destruction is the useful work that is destroyed due to irreversibilities in a process [11].

Thermochemical properties, including absolute entropies ($S_{0}$) for all isomers up to C_10_ at temperatures ranging from 0 to 1500 K, have been reported by Scott [12]. Experimental data for the thermochemical properties of various alkanes are available in the literature and can be accessed through the NIST Chemistry Webbook [13]. However, there are limited experimental data for alkanes longer than C_10_. Several group contribution methods exist in the literature to compute thermochemical properties, which includes those by Benson et al. [14], Constantinou and Gani [15], Joback and Reid [16], Marero and Gani [17], Hukkerikar et al. [18], Albahri and Aljasmi [19], and Domalski and Hearing [20]. Yaw’s Handbook [21] and the Design Institute for Physical Properties (DIPPR) database [22] also list thermochemical properties for many long-chain alkanes, which are either obtained from experiments or group contribution methods. Machine Learning (ML) models have emerged as an alternative for predicting thermochemical properties. Aldosari et al. successfully used Support Vector Regression (SVR), ν-SVR, and Random Forest Regression (RFR) algorithms to predict $\Delta_{f}S_{0}$, $\Delta_{f}H_{0}$, and $c_{p,0}$ for hydrocarbons [1]. For longer hydrocarbons, High-Dimensional Model Representation (HDMR) could be considered as another approach to predict these thermochemical properties [23].

Most of the group contribution methods in the literature are often inaccurate because these methods only consider first-order groups (CH_3_, CH_2_, CH, and C) or combine with a few second-order groups [15,24]. Second-order group contributions consider interactions between neighboring groups of atoms [25]. Increasing the number of second-order groups improves the predictions of thermochemical properties because these groups also account for interactions between the nearest neighboring groups of atoms [25]. Scott’s correlation [26], based on statistical mechanics, provides accurate predictions till C_10_ isomers when compared to experiments. However, this correlation is complex and difficult to apply to long-chain alkanes (>$C_{10}$), as it requires numerous functions and fitting parameters. While Machine Learning models offer promising predictive power, these models may struggle with extrapolation for long-chain alkanes [1,27]. In such cases, linear regression (LR) can outperform ML models when the independent variables are accurate and the output has a linear relationship with these variables [27].

In our recent work, we developed a user-friendly linear regression (LR) model based on second-order group contributions to predict thermochemical properties like Gibbs free energies $\left(G_{0}-H_{0}\left(0\;K\right)\right)$, enthalpies $\left(H_{0}-H_{0}\left(0\;K\right)\right)$, Gibbs free energies of formation $\Delta_{f}G_{0}$, and enthalpies of formation $\Delta_{f}H_{0}$ for alkanes longer than C_10_ for temperatures in the range (0–1000) K [25]. The second-order groups consider the interactions between the central atom and the neighboring groups. These groups are solely determined based on the topology of the alkanes with SMILES strings as input. Therefore, no 3D structures of alkanes were used in our LR model. Here, we calculated absolute entropies $S_{0}$ from $\left(G_{0}-H_{0}\left(0\;K\right)\right)$ and $\left(H_{0}-H_{0}\left(0\;K\right)\right)$, predicted using the LR model. The entropy values are in excellent agreement with both Scott’s data [12] and experimental values [26]. Our method effectively captures the variations in entropy in isomers due to differences in branching. The $S_{0}$ values are used to compute the entropy production for the hydroisomerization of alkanes in different zeolites at reaction equilibrium. Understanding the reaction product distribution at equilibrium is key to optimizing branched isomer yield in hydroisomerization [28]. Hydroisomerization involves the adsorption and subsequent dehydrogenation of linear alkanes at the metal sites of zeolites, forming alkenes [29]. These alkenes are protonated at the acid sites, resulting in the formation of alkyl carbenium ions [30]. Alkanes are regenerated from these alkylcarbenium ions via hydrogenation [30]. Hydroisomerization is accompanied by cracking reactions depending on the operating temperature of the reactor. This study is relevant for conditions where the hydroisomerization reaction approaches chemical equilibrium with a negligible amount of cracked products. Cracking reactions, being irreversible in nature [31], must be excluded from the reaction equilibrium distribution study. Calculating reaction equilibrium distributions for hydroisomerization reactions also provides insights into the shape selectivity effects of zeolites during these processes [32]. The calculation of entropy production in an equilibrium reactor involves steady-state mass and energy balances. The reaction equilibrium distribution provides the amount of isomers at the reactor outlet, while the energy balance determines the heat input required for the reactions. We analyzed the heat input and the entropy production for C_7_ isomers in FAU-, ITQ-29, BEA-, MEL-, MFI-, MTW-, and MRE-type zeolites at 500 K, observing small variations in these zeolites due to differences in reaction equilibrium distributions. Both heat input and entropy production increase with longer alkanes and higher temperatures, as the enthalpies and the absolute entropies increase with increasing chain length and temperature.

This article is organized as follows: Section 2 presents the key equations for computing the absolute entropies of alkanes, the mass balance in the equilibrium reactor, the energy balance for calculating the heat input, and the entropy balance for determining the entropy production. Section 3 discusses the results, showing that the computed entropies are highly accurate. A minimal variation in entropy production and heat input is observed for different zeolites during hydroisomerization of linear alkanes. Both heat input and entropy production increase with longer alkane chains and higher temperatures. Section 4 provides conclusions on the accuracy and usefulness of the entropy data for alkanes and the effects of zeolites, chain length, and temperature on entropy production for hydroisomerization at reaction equilibrium. This article also includes Supporting Information SI.xlsx, which contains the computed entropy values derived from the enthalpies and Gibbs free energies predicted by our LR model for isomers ranging from C_1_ to C_14_. SI.xlsx also contains the comparison between the absolute entropies computed using our model [25] and those predicted using group contribution methods by Benson et al. [14] and Constantinou and Gani [15].

## 2. Theory

**Figure 1:**
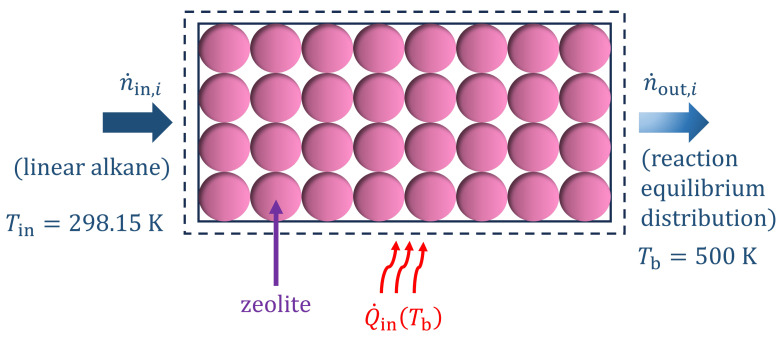
Schematic representation of a reactor for the hydroisomerization of linear alkanes at chemical equilibrium. The feed at the column inlet $\left({\dot{n}}_{\mathrm{in},i}\right)$ is at 298.15 K. The heat input $\left({\dot{Q}}_{\mathrm{in},i}\right)$ and the reactions take place at 500 K.

The absolute entropy $S_{0}$ is computed as follows [3]

(1) $$S_{0}=\left(\frac{1}{T}\right)\left[\left(H_{0}-H_{0}\left(0\;K\right)\right)-\left(G_{0}-H_{0}\left(0\;K\right)\right)\right]$$

where $\left(H_{0}-H_{0}\left(0\;K\right)\right)$ is the enthalpy at temperature *T* relative to 0 K. 0 K is considered as the reference temperature in this study. $\left(G_{0}-H_{0}\left(0\;K\right)\right)$ is the Gibbs free energy at temperature *T* relative to 0 K. These thermochemical properties are predicted using our LR model based on second-order group contributions for hydrocarbons [25]. The computed absolute entropies $S_{0}$ for alkane isomers $\left(C_{1}-C_{14}\right)$ at temperatures (0–1000) K are provided in the Supporting Information SI.xlsx. $S_{0}$ values are used to calculate the entropy production in a hydroisomerization reactor at reaction equilibrium (Figure 1), where ideal gas behavior is assumed. Calculating the entropy production requires molar flow rates at the inlet and the outlet of the reactor and the rate of heat input. Both heat input and entropy production are computed per unit moles of fluid mixture at the inlet. A steady-state molar balance is considered in the reactor as we assume that all isomerization reactions are at equilibrium:

(2) $$\sum_{i=1}^{N_{\mathrm{comp}}}{\dot{n}}_{\mathrm{in},i}=\sum_{i=1}^{N_{\mathrm{comp}}}{\dot{n}}_{\mathrm{out},i}$$

where ${\dot{n}}_{\mathrm{in},i}$ and ${\dot{n}}_{\mathrm{out},i}$ are the molar flow rates of component *i* at the reactor column inlet and outlet, respectively. At the inlet, only the linear alkane is considered at 298.15 K. The composition at the outlet is obtained from the reaction equilibrium distribution for the hydroisomerization of linear alkanes at infinite dilution in the zeolite. The reaction equilibrium distribution is obtained by imposing a gas-phase reaction equilibrium for the alkane isomers and a simultaneous phase equilibrium between the gas and the adsorbed phase for each component [32]. This satisfies the reaction equilibrium distribution in the zeolites [33]. A similar approach was also adopted by Hansen et al. [34] to study the influence of silicalite-1 pores on the reaction equilibrium distribution of the propene metathesis reaction. The adsorbed phase loadings for these isomers are obtained using Henry’s law. The Henry coefficients are computed using Widom’s test particle insertion method [35,36] in the RASPA software [37,38,39]. The mole fractions in the adsorbed phase depends on the Henry coefficients and the gas-phase mole fractions. The total pressure cancels out from the equation [32]. For further details on this method, the reader is referred to Refs. [25,32]. The gas-phase reaction equilibrium distribution is computed using $\left(G_{0}-H_{0}\left(0\;K\right)\right)$ at the operating temperature *T* and $\Delta_{f}H_{0,i}\left(0\;K\right)$ at 0 K [32]. For alkanes longer than C_10_ isomers, these properties are obtained using our LR model [25]. The rate of heat input ${\dot{Q}}_{\mathrm{in}}$ to the reactor is computed from the steady-state energy balance for the reactor [3]:

(3) $${\dot{Q}}_{\mathrm{in}}=\sum_{i=1}^{N_{\mathrm{comp}}}{\dot{n}}_{\mathrm{out},i}H_{i}-\sum_{i=1}^{N_{\mathrm{comp}}}{\dot{n}}_{\mathrm{in},i}H_{i}$$

where $H_{i}$ is the enthalpy of component *i*, which is computed as follows [3]:

(4) $$H_{i}\left(T\right)=\Delta_{f}H_{0,i}\left(0\;K\right)+\left(H_{i}\left(T\right)-H_{i}\left(0\;K\right)\right)$$

In Equation (4), $\Delta_{f}H_{0,i}\left(0\;K\right)$ is the enthalpy of formation of component *i* at 0 K. The entropy production $\sigma_{\mathrm{production}}$ is computed using the steady-state entropy balance for the reactor [3]:

(5) $$\sigma_{\mathrm{production}}=\sum_{i=1}^{N_{\mathrm{comp}}}{\dot{n}}_{\mathrm{out},i}S_{\mathrm{out},i}-\sum_{i=1}^{N_{\mathrm{comp}}}{\dot{n}}_{\mathrm{in},i}S_{\mathrm{in},i}-\frac{{\dot{Q}}_{\mathrm{in}}}{T_{b}}$$

$S_{\mathrm{in},i}$ and $S_{\mathrm{out},i}$ in Equation (5) are the absolute entropies of component *i* at the inlet and the outlet of the reactor. $S_{\mathrm{in}/\mathrm{out},i}$ depends on the fluid composition at the inlet and outlet of the reactor [3]. The heat input and the reactions take place at temperature $T_{b}$.

(6) $$S_{\mathrm{in}/\mathrm{out},i}=S_{0,i}-R\ln\left(\frac{x_{\mathrm{in}/\mathrm{out},i}P}{P_{\mathrm{ref}}}\right)$$

where $S_{0,i}$ is the absolute entropy for an isolated molecule of component *i*, *R* is the universal gas constant, and $x_{\mathrm{in}/\mathrm{out},i}$ are the mole fractions of component *i* at the inlet or outlet of the reactor. *P* is the operating pressure of the reactor and $P_{\mathrm{ref}}$ is the reference pressure, which is considered as 1 bar in this study. The term $R\ln\left(P/P_{\mathrm{ref}}\right)$ drops out in the entropy balance (Equation (5)) because the total molar flow rates at the inlet and outlet are equal (Equation (2)). For all cases, the operating temperature $T_{b}$ is 500 K.

## 3. Results and Discussion

**Figure 2:**
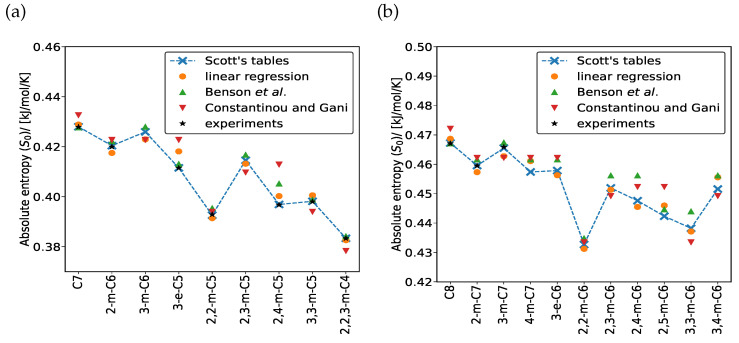
Computation of absolute entropies $S_{0}$ for (**a**) C_7_ and (**b**) C_8_ isomers at 298.15 K using our LR model, Benson et al.’s group additivity method [14], Constantinou and Gani’s group contribution [15], Scott’s tables [12], and the experimental data listed by Scott [26]. The predictions using the LR model are in excellent agreement with Scott’s tables and the data from the experiments [26]. The dashed blue line through the data points obtained from Scott’s tables is a guide to the eye.

Figure 2 compares the absolute entropies of C_7_ (Figure 2a) and C_8_ (Figure 2b) isomers at 298.15 K, calculated using our LR model [25], the group contribution methods of Benson et al. [14] and Constantinou and Gani [15], and data obtained from Scott’s tables [12] and experimental results [26]. The data in the Scott’s tables were obtained by fitting statistical mechanics-based correlations to experimental measurements [26]. The entropies predicted by our LR model [25] show excellent agreement with both Scott’s tables and experimental data. Unlike the group additivity method by Benson et al. [14] and the group contribution approach by Constantinou and Gani [15], our LR model successfully distinguishes between different isomers based on the number, types and positions of the branches. The Mean Absolute Errors (MAEs) of $\left(H_{0}-H_{0}\left(0\;K\right)\right)$ and $\left(G_{0}-H_{0}\left(0\;K\right)\right)$, predicted by our linear regression model, are 1.012 kJ/mol and 0.181 kJ/mol, respectively [25]. The MAE for the product of temperature and absolute entropy $S_{0}$ is 1.03 kJ/mol, which exceeds the chemical accuracy of 4.184 kJ/mol. The dataset comparing the absolute entropies computed by our method and those predicted by group contribution methods of Benson et al. [14] and Constantinou and Gani [15] for C_14_ isomers at 298.15 K is included in the excel worksheet S0_comparison of the Supporting Information SI.xlsx.

**Figure 3:**
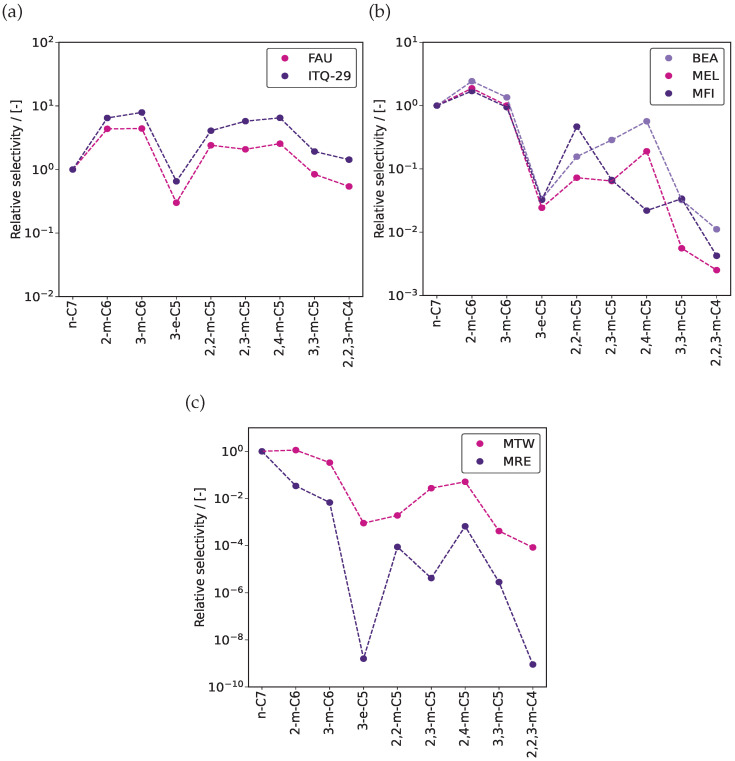
Selectivities of C_7_ isomers relative to n-C_7_ at reaction equilibrium in (**a**) FAU- and ITQ-29-type zeolites, (**b**) BEA-, MEL-, and MFI-type zeolites, and (**c**) MTW- and MRE-type zeolites at infinite dilution and 500 K. The absolute selectivity is defined as the mole fraction of a certain component divided by the sum of the mole fractions of all other components [41]. The relative selectivity refers to the ratio of the absolute selectivity of a specific isomer to that of the reference isomer, which is C_7_ in this case. The dashed lines through the data points are a guide to the eye.

The composition at the reactor column outlet represents the reaction equilibrium distribution for the hydroisomerization of alkanes in different zeolites at infinite dilution. Figure 3 shows the reaction equilibrium distributions for the hydroisomerization of C_7_ isomers in FAU-, ITQ-29-, BEA-, MEL-, MFI-, MTW-, and MRE-type zeolites at 500 K. These zeolites are classified into three categories based on the shape and size of the pores, which show distinct selectivity for alkanes. FAU- and ITQ-29-type zeolites have cage-like pore structures [40]. BEA-, MEL-, and MFI-type zeolites feature three-dimensional channels with intersections [40]. MTW- and MRE-type zeolites contain one-dimensional pore structures [40]. The datasets for the reaction equilibrium distributions are included in the excel sheet xi_C7_500K of the Supporting Information SI.xlsx. In FAU- and ITQ-29-type zeolites (Figure 3a), the adsorbed phase selectivity is primarily influenced by the gas-phase thermochemical properties $\Delta_{f}H_{0}\left(0\;K\right)$ and $\left(G_{0}-H_{0}\left(0\;K\right)\right)$. Dimethyl isomers $\left({2,2\text{-}m\text{-}C}_{5},\;{2,3\text{-}m\text{-}C}_{5},\;\mathrm{and}\;{2,4\text{-}m\text{-}C}_{5}\right)$ are favored compared to n-C_7_ inside these zeolites. Due to larger pore diameters, the influence of pore structure on selectivities is small. FAU- and ITQ-29-type zeolites can accommodate molecules as large as 11.24 Å and 11.05 Å, respectively [40]. BEA-, MEL-, and MFI-type zeolites with 3D channel-like pores connected via intersections show similar selectivities for n-C_7_ and the monomethyl isomers $\left({2\text{-}m\text{-}C}_{6}\;\mathrm{and}\;{3\text{-}m\text{-}C}_{6}\right)$, which are preferentially formed compared to dimethyl-, trimethyl-, and ethyl-branched isomers (Figure 3b). In BEA-, MEL-, and MFI-type zeolites, variations in selectivities are influenced by both gas-phase thermochemical properties and Henry coefficients. In MTW- and MRE-type zeolites, n-C_7_ has the highest selectivity, followed by 2-m-C_6_ and 3-m-C_6_, with variations influenced largely by the Henry coefficients (Figure 3c) because of the smaller diameters and one-dimensional nature of the pores [32]. The overall differences in selectivities between the linear and the branched isomers are mainly determined by the pore diameters of the zeolites. For isomers with an identical degree of branching, the selectivities are determined by the distributions and the shapes of the pore structures.

**Figure 4:**
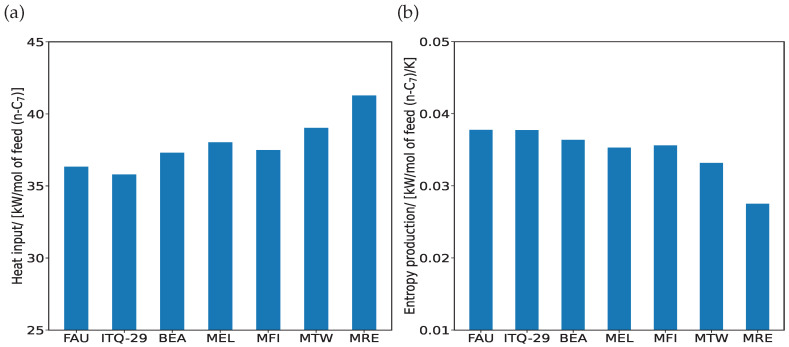
Variations of (**a**) heat input ${\dot{Q}}_{\mathrm{in}}$ and (**b**) entropy production $\sigma_{\mathrm{production}}$ for the hydroisomerization of C_7_ isomers at reaction equilibrium in FAU-, ITQ-29-, BEA-, MEL-, MFI-, MTW-, and MRE-type zeolites. The reactions take place at 500 K and infinite dilution in the zeolite.

Figure 4 shows the variations in the rates of heat input (Figure 4a) and entropy production (Figure 4b) for the hydroisomerization of C_7_ isomers in an equilibrium reactor for different zeolites. Both heat input and entropy production show small variations with the zeolites, influenced by the unique reaction equilibrium distributions in each type of these zeolites. The rate of heat input slightly increases from large pore zeolites (FAU- and ITQ-29-types) to narrower 1D zeolites (MTW- and MRE-types) (Figure 4a). This is because n-C_7_, 2-m-C_6_, and 3-m-C_6_ isomers which have higher enthalpies compared to the multi-branched isomers are formed in large proportions in these 1D zeolites at reaction equilibrium. The entropy production decreases with decreasing pore size of the zeolites due to smaller values of entropies for these linear and mono-branched alkanes at reaction equilibrium in these zeolites. This may vary when cracking reactions are involved.

**Figure 5:**
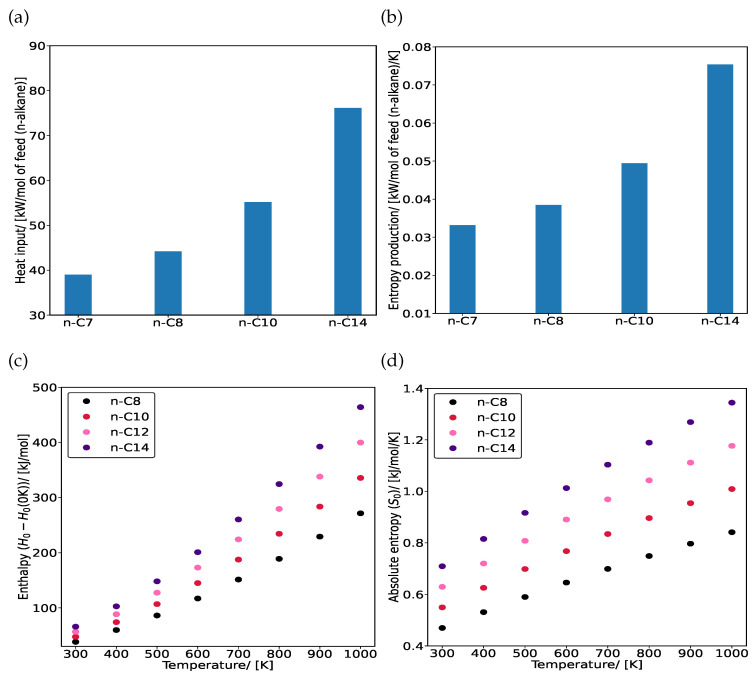
Effect of chain length on (**a**) heat input ${\dot{Q}}_{\mathrm{in}}$ and (**b**) entropy production $\sigma_{\mathrm{production}}$ for the hydroisomerization of C_7_, C_8_, C_10_, and C_14_ isomers at reaction equilibrium in MTW-type zeolite. The reactions take place at 500 K and infinite dilution. Variations of (**c**) enthalpies $H_{0}-H_{0}\left(0\;K\right)$ and (**d**) absolute entropies $S_{0}$ with temperatures in the range (300–1000) K for n-C_8_, n-C_10_, n-C_12_, and n-C_14_ isomers.

The hydroisomerization of linear alkanes n-C_7_, n-C_8_, n-C_10_, and n-C_14_ is studied in MTW-type zeolite at 500 K and infinite dilution. With increasing chain length, both heat input to the reactor (Figure 5a) and entropy production (Figure 5b) increase because of the rapid increase in enthalpies and entropies with temperature for long-chain alkanes, as shown in Figure 5c,d, respectively. The enthalpies increase with chain length due to the larger number of carbon–hydrogen (C-H) and carbon–carbon (C-C) bonds, each adding to the total enthalpy of the molecule. Longer alkanes also have higher absolute entropies compared to the shorter ones because the larger number of atoms allows for more molecular arrangements [42]. This leads to an increase in entropy production during the hydroisomerization of longer alkanes.

**Figure 6:**
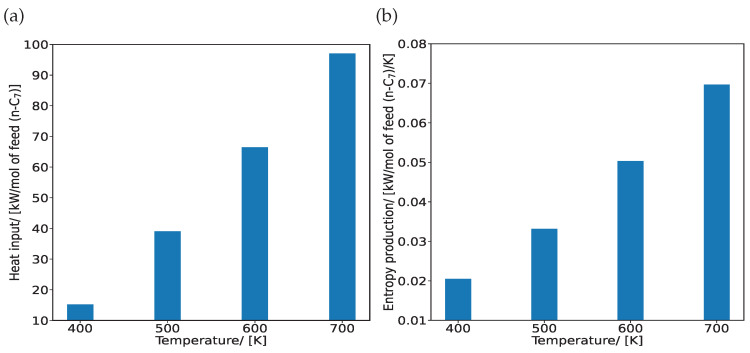
Effect of temperature on (**a**) heat input ${\dot{Q}}_{\mathrm{in}}$ and (**b**) entropy production $\sigma_{\mathrm{production}}$ for the hydroisomerization of C_7_ isomers at reaction equilibrium in MTW-type zeolite at 400, 500, 600, and 700 K. The reactions take place in the zeolite at infinite dilution.

Figure 6a shows an increase in the rate of heat input with increasing temperature. The internal energy increases at higher temperatures, which leads to higher enthalpy values. This increases the rate of heat requirement in the reactor column. The absolute entropies of alkanes increase with temperature because higher temperatures provide more energy to the molecules to occupy a larger number of configurations. This leads to an increase in the rate of entropy production with temperature (Figure 6b).

## 4. Conclusions

The absolute entropies ($S_{0}$) of alkanes computed from $\left(H_{0}-H_{0}\left(0\;K\right)\right)$ and $\left(G_{0}-H_{0}\left(0\;K\right)\right)$ as predicted by our LR model show excellent agreement with Scott’s tables [12] and experimental data [26]. Our model effectively captures entropy variations based on the type, number, and positions of branches in isomers, outperforming group contribution methods like those by Benson et al. [14] and Constantinou and Gani [15]. The computed absolute entropies will be valuable for designing and optimizing alkane-based processes with a focus on second law efficiency. Unique reaction product distributions are observed in the hydroisomerization of C_7_ isomers in different zeolites. Large-pore zeolites, such as FAU- and ITQ-29-type zeolites, favor dimethyl isomers compared to n-C_7_, which is mainly due to $\left(G_{0}-H_{0}\left(0\;K\right)\right)$ and $\Delta_{f}H_{0}\left(0K\right)$. In BEA-, MEL-, and MFI-type zeolites, normal alkanes and monomethyl isomers show similar selectivities with larger preferences compared to dimethyl and trimethyl isomers for C_7_ and C_8_ alkanes. In narrow-pore zeolites (MRE- and MTW-types), n-C_7_ is preferred, followed by mono-branched isomers. Small variations in heat input and entropy production are observed for different zeolites because of the unique reaction product distributions, which are influenced by the shapes and sizes of the zeolite pores. These trends may shift when cracking reactions are considered. As the alkane chain length increases, both heat input and entropy production increase. This is due to the increase in enthalpies and entropies with temperature for longer chains. A larger number of C-H and C-C bonds in long-chain alkanes increases the internal energy, leading to a larger enthalpy and, consequently, higher heat input. The increased number of atoms in long-chain alkanes allows for more molecular conformations, leading to higher absolute entropies. At higher temperatures, both heat input and entropy production for the hydroisomerization of a certain alkane (C_7_ in this case) increase. The rate of heat input increases because of the larger enthalpies of the isomers at higher temperatures. At higher temperatures, molecules gain more energy to occupy a larger number of configurations, causing an increase in entropy and therefore an increase in entropy production. This work demonstrates the accuracy of our LR model in predicting absolute entropies of alkanes and offers insights into heat requirements and entropy production during the formation of branched isomers in zeolite-catalyzed hydroisomerization, which will be useful for better design and optimization of this process. The computed entropies will also be relevant for improving energy efficiencies in other processes involving hydrocarbons. 

## Data Availability

The raw data supporting the conclusions of this article are available in the Supporting Information SI.xlsx.

## Supplementary Materials

The following supporting information can be downloaded at: https://www.mdpi.com/article/10.3390/e26121120/s1.
